# Expected population weight and diabetes impact of the 1-peso-per-litre tax to sugar sweetened beverages in Mexico

**DOI:** 10.1371/journal.pone.0176336

**Published:** 2017-05-17

**Authors:** Tonatiuh Barrientos-Gutierrez, Rodrigo Zepeda-Tello, Eliane R. Rodrigues, Arantxa Colchero-Aragonés, Rosalba Rojas-Martínez, Eduardo Lazcano-Ponce, Mauricio Hernández-Ávila, Juan Rivera-Dommarco, Rafael Meza

**Affiliations:** 1 Centre for Population Health Research, National Institute of Public Health, Cuernavaca, Mexico; 2 Instituto de Matemáticas, Universidad Nacional Autónoma de México, Mexico City, Mexico; 3 Centre for Health Systems Research, National Institute of Public Health, Cuernavaca, Mexico; 4 General Direction, National Institute of Public Health, Cuernavaca, Mexico; 5 Centre for Nutrition and Health Research, National Institute of Public Health, Cuernavaca, Mexico; 6 Department of Epidemiology, School of Public Health, University of Michigan, Ann Arbor, United States of America; Cinvestav-Merida, MEXICO

## Abstract

**Study question:**

What effect on body mass index, obesity and diabetes can we expect from the 1-peso-per-litre tax to sugar sweetened beverages in Mexico?

**Methods:**

Using recently published estimates of the reductions in beverage purchases due to the tax, we modelled its expected long-term impacts on body mass index (BMI), obesity and diabetes. Microsimulations based on a nationally representative dataset were used to estimate the impact of the tax on BMI and obesity. A Markov population model, built upon an age-period-cohort model of diabetes incidence, was used to estimate the impact on diagnosed diabetes in Mexico. To analyse the potential of tax increases we also modelled a 2-peso-per-litre tax scenario.

**Study answer and limitations:**

Ten years after the implementation of the tax, we expect an average reduction of 0.15 kg/m^2^ per person, which translates into a 2.54% reduction in obesity prevalence. People in the lowest level of socioeconomic status and those between 20 and 35 years of age showed the largest reductions in BMI and overweight and obesity prevalence. Simulations show that by 2030, under the current implementation of 1-peso-per-litre, the tax would prevent 86 to 134 thousand cases of diabetes. Overall, the 2-peso-per-litre scenario is expected to produce twice as much of a reduction. These estimates assume the tax effect on consumption remains stable over time. Sensitivity analyses were conducted to assess the robustness of findings; similar results were obtained with various parameter assumptions and alternative modelling approaches.

**What this study adds:**

The sugar-sweetened beverages tax in Mexico is expected to produce sizable and sustained reductions in obesity and diabetes. Increasing the tax could produce larger benefits. While encouraging, estimates will need to be updated once data on direct changes in consumption becomes available.

## Introduction

Changes in dietary patterns and physical activity have led to a historical increase in body weight and metabolic diseases. Worldwide, the proportion of adults with a body mass index (BMI) of 25 kg/m^2^ or higher increased between 1980 and 2013 from 28.8% to 36.9% in men, and from 29.8% to 38.0% in women.[1] Excess body weight is linked to major chronic diseases such as diabetes, cardiovascular disease, and cancer.[2] Currently, achieving population weight reduction is considered the single most important public health goal after tobacco control.[3]

Diabetes and obesity are fundamental public health problems in Mexico. In 2012, 71% of the adult population was overweight or obese,[4] and 9.2% had been diagnosed with diabetes.[5] High fasting plasma glucose, excessive BMI, and dietary risks account for more than 30% of disability-adjusted life years and over 50% of the deaths in the country.[6] In 2008 the total cost of obesity-related diseases (type 2 diabetes, cardiovascular disease, breast cancer and osteoarthritis) was 4 billion USD per year.[7] Considering the large toll of excess weight and diabetes, calls to implement nationwide efforts to reduce obesity and diabetes in the country have been made.[7,8]

Sugar sweetened beverages (SSB) are one of the largest sources of calories with no added nutritional value.[9] SSB consumption has been associated with increased weight, type-2 diabetes, and other diseases.[2,10–13] In Mexico, SSB contribute 9.8% of the total caloric intake of children and adults,[14] and 70.3% of the total added sugar in the diet.[15] Increasing the price of SSB through taxation has been proposed as a public health intervention to reduce caloric intake and population weight, and to address the increasing burden of obesity and diabetes.[16,17]

In January 2014 the Mexican government implemented a 1 peso-per-litre excise tax to all non-alcoholic beverages with added sugar, which represents an approximate 10% increase in shelf-price. Using purchases data, a recent study attributed an average 6.1% reduction in SSB consumption to the tax.[18] Studies have projected the expected impact of a hypothetical SSB tax to be implemented in India, South Africa, Ireland, UK, Australia and the USA. [19–24] However, none of these countries have yet implemented a nation-wide SSB tax. Out of the Mexican experience a recent paper estimated the expected effect of the tax on cardiovascular disease and diabetes, assuming a 10% reduction in consumption for the adult population;[25] however, to date no projections have been done using the observed effect of the tax, as recently calculated by Colchero, et al., while also considering differential effects by socioeconomic status (SES) and evaluating its potential effect on obesity and body weight.[18] We aimed to estimate the expected impact of the tax on body weight and on the prevalence of overweight, obesity and diabetes in Mexico.

## Methods

### Model overview

To assess the impact of the SSB tax on population weight and diabetes, we constructed a simulation model, summarized as follows (S1 Fig in S1 File): 1) we obtained pre-tax information on SSB consumption from a nationally-representative survey, 2) we applied the percent change in SSB purchases attributable to the tax to baseline SSB consumption to obtain the expected volume change in SSB consumption, 3) we transformed volume into caloric change and introduced it into a dynamic weight change model to obtain the expected change in body weight, obesity and overweight prevalence, 4) using a Markov Model for diabetes in Mexico, we modified the diabetes risk according to the change in SSB consumption to obtain the expected change in diabetes cases and prevalence. A hypothetical 20% tax was also modelled using a proportional shift in consumption.

#### Data

The main data source for the models was the 2012 National Health and Nutrition Survey (ENSANUT from its Spanish acronym).[26] ENSANUT-2012 was a complex, nationally-representative survey involving 45,000 households (96,031 individuals).[27] This survey included demographic, socioeconomic, nutritional, and health-related information through questionnaires applied by trained field workers to selected individuals in a random sample of households. Detailed anthropometric and dietary intake data is obtained from a subsample of participants. All analyses were restricted to individuals 20-years old and older, with complete anthropometric and nutritional data (2,735 that expand to 63,151,429 individuals). Sampling weights and sample design were considered in all procedures requiring population-level estimates.

#### Pre-tax SSB consumption

Pre-tax SSB consumption was obtained from the ENSANUT-2012 food frequency questionnaire. SSB were selected to match those considered in the 1-peso-per-litre tax: *“*… *all sugar sweetened and flavoured beverages; concentrates*, *powders*, *syrups*, *scents and extracts that once reconstituted become flavoured beverages; and syrups or concentrates used to prepare flavoured beverages to be sold in open containers*…*”*.[28] We included sugar-sweetened carbonated SSB, industrialized juices, and industrialized *aguas frescas* (traditional SSB). SSB prepared at home or at restaurants were excluded from this group, as the tax should not affect homemade preparations. All types of SSB were summed up to obtain the total SSB consumption per individual. Population SSB consumption quartiles were constructed, following common methods of SSB analysis,[9,29] being: Q1 (≤33.94 ml/person/day), Q2 (33.94–129.64 ml/person/day), Q3 (129.64–402.86 ml/person/day), and Q4 (≥402.86 ml/person/day).

#### Tax-related change in SSB

Percent reductions in SSB consumption attributable to the tax were obtained from *Colchero et al*.[18] Using data from January to December 2014, they estimated an average 6.1% decrease in SSB purchases (hereafter “average tax effect”), varying by socioeconomic status (SES), (*low*: -9.1%, *medium*: -5.6%, *high*: -5.5%). Reductions reached their peak level in December 2014, with an estimated 11.9% decrease (hereafter “peak monthly effect”), varying by socioeconomic status (*low*: -17.4%, *medium*: -13.1%, *high*: -6.8%); we selected these values to model an alternative “best case” scenario. For the hypothetical 20% tax scenario, we assumed twice the average and peak monthly effects.

#### Anthropometry

Body weight (kilograms) and height (meters) were directly measured using standardized procedures and instruments.[27] BMI was calculated as weight divided by the square of height and classified according to the WHO into normal (BMI < 25 kg/m^2^), overweight (BMI ≥ 25 & < 30 kg/m^2^) or obese (BMI ≥ 30 kg/m^2^).[30]

#### Demographics

Sex and age were self-reported. Mexican adults tend to consume less SSB as they age,[31] so we divided the groups in young adults (20 to 40 years-old), adults (40 to 60 years-old), and elderly (60 years and more). Socioeconomic status was obtained from the socioeconomic status index of the ENSANUT-2012 and divided into tertiles.[32]

#### Diagnosed diabetes

Diagnosed diabetes was assessed across ENSANUT survey years with the question: “*Has a physician ever told you that you had high blood sugar or diabetes*?”. Time since diagnosis was obtained from the question *“How long has it been since a physician told you for the first time you had high blood sugar or diabetes*?*”*.

#### Age-specific mortality

Age-specific mortality rates by sex for 2010 were obtained from the National System of Health Information (SINAIS).[33]

#### Population projections

Projected births by sex and calendar year (2010–2050) for the Mexican population were obtained from the National Population Council of Mexico (CONAPO).[34]

### Models

#### Expected change in consumption

The expected change in SSB consumption was estimated for all individuals considering their SES, multiplying each individual SSB consumption by the change in SSB purchases estimated by *Colchero*, *et al*. for 2014 (average and peak monthly effect). We assumed the reduction occurred at the beginning of the first year and remained constant throughout time.

#### Weight change model

To estimate the change in weight due to the tax we used the model developed by Chow and Hall, which has been validated with experimental weight change data,[35–38] and has been frequently used to estimate the potential impact of SSB taxes.[19,22,39] Briefly, the model estimates the body weight of an individual at time *t*, considering changes in extra cellular fluid, glycogen, and fat and lean tissues. A full description of the model can be found in the S2 File Section 1.

Sex, age, weight and height for ENSANUT-2012 participants were used to initialize the model and to obtain intermediate variables, such as resting metabolic rate. We assumed that all participants were sedentary (physical activity level of 1.5). The model estimates short- and long-term changes; we chose to present the change after 10-years as the final measure of effect.

#### Diabetes model

We used a multi-cohort Markov state-transition model of diagnosed diabetes to project the evolution of diabetes rates in the country (S2 Fig in S1 File).[40] Briefly, the model considers estimations of age-specific incidence of diagnosed diabetes (from ENSANUT), age-specific mortality rates (from SINAIS) and projected birth rates (from CONAPO) to produce scenarios for future age-specific incidence and prevalence of diagnosed diabetes. Further details can be found in S2 File Section 2.

Mexico has experienced a considerable surge in diabetes in the recent years, thus, we assumed three scenarios for future age-specific incidence of diagnosed diabetes, corresponding to those observed in the last 10 years: optimistic (2000), intermediate (2005), and pessimistic (2010).[40] To assess the effect of the tax we compared the baseline diabetes prevalence projection from 2015–2050 to a counterfactual scenario. The counterfactual assumes that the Mexican population will reduce their SSB consumption according to either the average or the peak monthly effect stratified by SES tertiles. Diabetes and SSB consumption were linked using the meta-analytical relative risk (RR) of 1.26 per SSB serving (*RR* 1.26, 95% *CI* 1.12, 1.41) estimated by *Malik et al*.[11] This RR directly links SSB consumption to diabetes risk, including both direct and weight-mediated effects.

All analyses were conducted in R-statistical software (R version 3.3.0 (2016-05-03)) using the *deSolve* package.[41,42]

### Ethical approval

This study (No 1456–6988) is exempt from approval by internal review board (reviewed by National Institute of Public Health Ethics, Research and Biosecurity Committees).

## Results

Table 1 presents the baseline characteristics of the population by quartiles of SSB consumption. Overall, 30.38% had normal weight, 35.57% were overweight and 34.05% were obese. Obesity prevalence was higher among individuals in the third (36.43%) and fourth (36.94%) quartiles of SSB consumption. Females accounted for 55.13% of the expanded sample; the proportion of females was smaller in the highest quartiles of SSB consumption (66.5% in Q1, 43.2% in Q4). Participants younger than 40 years represented 47.21% of the sample, with 34.20% between 40 and 59 years, and 18.59% having 60 years and over. A larger proportion of young adults was observed among heavier consumers (58.96% in the fourth quartile).

**Table 1:** Characteristics of the adult Mexican population by quartiles of taxed sugar sweetened beverage (SSB) consumption. ENSANUT 2012.

			Quartiles^1^ of SSB^2^ consumption			
		Total^3^ (n = 2,735)	Q1 (n = 659)	Q2 (n = 744)	Q3 (n = 711)	Q4 (n = 621)
**BMI classification**^**4**^ **(%)**						
	*Normal*	30.38	31.48	29.12	28.04	33.07
	*Overweight*	35.57	36.99	39.69	35.53	29.99
	*Obese*	34.05	31.53	31.19	36.43	36.94
**Sex (%)**						
	*Male*	44.87	33.46	39.00	49.49	56.82
	*Female*	55.13	66.54	61.00	50.51	43.18
**Age category in years (%)**						
	*20 - < 40*	47.21	31.41	45.53	51.76	58.96
	*40 - < 60*	34.20	40.37	34.52	32.99	29.39
	*60* ≤	18.59	28.22	19.95	15.25	11.65
**Socioeconomic status (%)**						
	*Low*	33.65	39.27	35.32	28.12	32.40
	*Medium*	32.91	28.00	30.73	38.36	34.16
	*High*	33.43	32.73	33.96	33.52	33.45

^1^ Taxed sugar sweetened beverages quartile categories (ml/person/day): Q1: ≤33.94, Q2: 33.94–129.64, Q3: 129.64–402.86, Q4: ≥ 402.86.

^2^ Sugar sweetened beverages (SSB) includes: carbonated beverages, *aguas frescas* and juices (industrialized).

^3^ Population expansion: Total: 63,151,429, Q1: 14,658,319, Q2: 16,469,474, Q3: 16,233,311, Q4: 15,790,324.

^4^ BMI cut-off points: Normal < 25, Overweight 25–29.99, Obese ≥ 30 kg/m^2^

Table 2 presents the average SSB consumption and caloric intake at baseline and the expected reduction after the implementation of the tax. The average SSB consumption was 323.49 ml/person/day, which translates into 125.50 kcal/person/day. Considering the average tax effect, SSB consumption was expected to decrease by 21.62 ml/person/day (8.38 kcal/person/day). The peak monthly tax effect would produce larger decreases in SSB consumption (40.18 ml/person/day, 15.56 kcal/person/day). Under the hypothetical 20% tax the average effect would produce a 43.23 ml/person/day reduction (16.75 kcal/person/day) and the peak monthly effect an 80.35 ml/person/day (31.13 kcal/person/day) reduction.

**Table 2:** Taxed sugar sweetened beverage (SSB) consumption in the adult Mexican population at baseline and expected reductions after the implementation of a 10% and 20% SSB tax-increase assuming the average and peak monthly observed sales-change for 2014 (n = 2,735)^1^.

Type	Baseline^2^	10% tax		20% tax	
		*Average*	*Peak Monthly*	*Average*	*Peak Monthly*
*Volume intake reduction (ml/person/day)*	323.49 (520.44)	-21.62 (36.12)	-40.18 (71.09)	-43.23 (72.23)	-80.35 (142.18)
*Caloric intake reduction (kcal/person/day)*	125.50 (198.80)	-8.38 (13.66)	-15.56 (26.87)	-16.75 (27.32)	-31.13 (53.74)

^1^ Expands to 63,151,429 individuals.

^2^ All values reported as: mean (SD).

Table 3 presents the expected BMI change 10 years after the implementation of the tax. For the overall population the starting BMI was 28.40 kg/m^2^, decreasing 0.15 kg/m^2^ under average and 0.29 kg/m^2^ under peak monthly tax effects (0.31 and 0.57 kg/m^2^ for the 20% tax). Baseline BMI was higher among high-SES participants, yet low-SES participants experienced larger BMI reductions than all others (0.20 kg/m^2^ and 0.37 kg/m^2^ for average and peak monthly effect, respectively). Younger adults showed the largest reduction in BMI; the 10% tax increase would induce a 0.18 kg/m^2^ reduction under average and 0.33 kg/m^2^ under peak monthly tax effect. BMI was similar across quartiles of SSB consumption; the tax had no effect in the first quartile of consumption, but it is expected to produce a reduction of 0.45 kg/m^2^ and 0.84 kg/m^2^ for the average and peak monthly effects among those in the fourth quartile (0.90 kg/m^2^ and 1.66 kg/m^2^ under a 20% tax). Similar patterns were observed for men and women, with the exception of SES: in men the tax produced larger BMI decreases in those of lower SES, yet in women the trend was small and only appeared for the average but not for the peak monthly effect. Fully stratified tables including initial BMI, expected BMI change at 1 and 10 years are included in (S1 File, Table S1 (all adults), Table S2 (males) and Table S3 (females)).

**Table 3:** Baseline body mass index (BMI) and expected change in the adult Mexican population 10 years after the implementation of a 10 and 20% tax to sugar sweetened beverages (SSB)^1^ assuming the average and peak monthly observed sale-changes of 2014.

		Total^2^					Men					Women				
		Baseline *kg/m*^*2*^	10% tax *kg/m*^*2*^ *(% change)*		20% tax *kg/m*^*2*^ *(% change*		Baseline *kg/m*^*2*^	10% tax *kg/m*^*2*^ *(% change)*		20% tax *kg/m*^*2*^ *(% change*		Baseline *kg/m*^*2*^	10% tax *kg/m*^*2*^ *(% change)*		20% tax *kg/m*^*2*^ *(% change*	
			*Average*	*Peak Monthly*	*Average*	*Peak Monthly*		*Average*	*Peak Monthly*	*Average*	*Peak Monthly*		*Average*	*Peak Monthly*	*Average*	*Peak Monthly*
**Total**		28.40	0.15 (0.55)	0.29 (1.01)	0.31 (1.08)	0.57 (2.00)	27.55	0.18 (0.66)	0.33 (1.21)	0.36 (1.30)	0.66 (2.40)	29.09	0.13 (0.46)	0.25 (0.85)	0.27 (0.92)	0.49 (1.69)
**Socioeconomic status**																
	*Low*	27.75	0.20 (0.71)	0.37 (1.34)	0.39 (1.40)	0.74 (2.66)	26.99	0.25 (0.94)	0.48 (1.78)	0.50 (1.86)	0.95 (3.51)	28.38	0.15 (0.52)	0.28 (1.00)	0.30 (1.04)	0.56 (1.98)
	*Medium*	28.74	0.14 (0.50)	0.33 (1.16)	0.28 (0.99)	0.66 (2.29)	27.43	0.15 (0.56)	0.36 (1.31)	0.31 (1.12)	0.71 (2.57)	29.77	0.13 (0.45)	0.31 (1.05)	0.27 (0.90)	0.62 (2.08)
	*High*	28.72	0.12 (0.44)	0.15 (0.54)	0.25 (0.87)	0.31 (1.07)	28.23	0.13 (0.47)	0.16 (0.58)	0.26 (0.93)	0.32 (1.15)	29.12	0.12 (0.41)	0.15 (0.51)	0.24 (0.81)	0.29 (1.01)
**Age (years)**																
	*20 - < 40*	27.83	0.18 (0.65)	0.33 (1.19)	0.36 (1.29)	0.65 (2.35)	27.40	0.22 (0.80)	0.40 (1.47)	0.43 (1.58)	0.80 (2.90)	28.14	0.15 (0.54)	0.28 (0.98)	0.30 (1.08)	0.55 (1.94)
	*40 - < 60*	29.59	0.15 (0.49)	0.28 (0.95)	0.29 (0.98)	0.56 (1.88)	28.16	0.17 (0.61)	0.33 (1.17)	0.34 (1.22)	0.65 (2.32)	30.77	0.12 (0.40)	0.24 (0.78)	0.25 (0.80)	0.48 (1.55)
	*60 ≤*	27.66	0.11 (0.38)	0.19 (0.67)	0.21 (0.76)	0.37 (1.34)	26.86	0.11 (0.42)	0.19 (0.71)	0.23 (0.84)	0.38 (1.41)	28.48	0.10 (0.35)	0.18 (0.64)	0.20 (0.69)	0.36 (1.27)
**SSB consumption**^3^																
	*Q1*	28.34	0.00 (0.01)	0.00 (0.01)	0.00 (0.01)	0.01 (0.02)	27.51	0.00 (0.00)	0.00 (0.00)	0.00 (0.00)	0.00 (0.01)	28.76	0.00 (0.01)	0.00 (0.01)	0.00 (0.01)	0.01 (0.02)
	*Q2*	28.24	0.04 (0.14)	0.07 (0.26)	0.08 (0.28)	0.15 (0.52)	27.13	0.04 (0.13)	0.06 (0.23)	0.07 (0.26)	0.12 (0.46)	28.96	0.04 (0.15)	0.08 (0.28)	0.09 (0.30)	0.16 (0.55)
	*Q3*	28.64	0.12 (0.41)	0.22 (0.77)	0.24 (0.83)	0.44 (1.54)	27.68	0.11 (0.39)	0.20 (0.72)	0.22 (0.78)	0.40 (1.44)	29.57	0.13 (0.44)	0.24 (0.82)	0.26 (0.87)	0.48 (1.63)
	*Q4*	28.37	0.45 (1.60)	0.84 (2.96)	0.90 (3.18)	1.66 (5.84)	27.75	0.45 (1.61)	0.83 (3.00)	0.89 (3.20)	1.64 (5.90)	29.19	0.46 (1.58)	0.85 (2.92)	0.92 (3.15)	1.69 (5.78)

1 Sugar sweetened beverages (SSB) includes: carbonated beverages, aguas frescas and juices (industrialized).

2 Population expansion: Total: 63,151,429, Q1: 14,658,319, Q2: 16,469,474, Q3: 16,233,311, Q4: 15,790,324.

3 Taxed sugar sweetened beverages quartile categories (ml/person/day): Q1: ≤33.94, Q2: 33.94–129.64, Q3: 129.64–402.86, Q4: ≥ 402.86.

Projected changes in the prevalence of overweight and obesity are presented in Table 4. At baseline 30.38% of participants had normal weight, 35.57% were overweight and 34.05% obese. After 10 years, under the average tax effect the simulations indicate that the prevalence of obesity would decrease by 2.54%, while overweight and normal weight would increase 0.51% and 2.25%, respectively. Under the peak monthly tax effect, obesity prevalence would decrease by 6.38%, while overweight (0.42%), and normal weight (6.66%) would increase. Larger decreases in obesity and overweight are expected for the 20% tax under average tax effect, with a net increase in the prevalence of normal weight of 6.90%, (12.95% under peak monthly tax effect). The impact of the tax increases with baseline SSB consumption: in the first quartile the impact is null, but in the fourth quartile the average tax effect is expected to decrease the prevalence of obesity by 7.33% (18.26% under peak monthly tax effect), and increase overweight (3.23% average, 1.98% peak monthly), and normal weight (5.25% average, 18.60% peak monthly).

**Table 4:** Baseline prevalence of overweight and obesity and expected change in the adult Mexican population 10 years after the implementation of a 10 and 20% tax to SSB^1^ assuming the average and peak monthly observed sale-changes of 2014.

Population	WHO categories^2^	Baseline prevalence	10% tax change prevalence (% change)		20% tax change prevalence (% change)	
			*Average*	*Peak Monthly*	*Average*	*Peak* *Monthly*
Total (n = 2,735)^3^	*Normal*	30.38	31.06 (2.25)	32.40 (6.66)	32.47 (6.90)	34.31 (12.95)
	*Overweight*	35.57	35.75 (0.51)	35.72 (0.42)	35.81 (0.67)	34.90 (-1.90)
	*Obese*	34.05	33.19 (-2.54)	31.88 (-6.38)	31.72 (-6.85)	30.80 (-9.57)
SSB Q1^4^ (n = 659)	*Normal*	31.48	31.48 (0.00)	31.48 (0.00)	31.48 (0.00)	31.48 (0.00)
	*Overweight*	36.99	36.99 (0.00)	36.99 (0.00)	36.99 (0.00)	36.99 (0.00)
	*Obese*	31.53	31.53 (0.00)	31.53 (0.00)	31.53 (0.00)	31.53 (0.00)
SSB Q2 (n = 744)	*Normal*	29.12	29.12 (0.00)	29.52 (1.39)	29.52 (1.39)	30.09 (3.35)
	*Overweight*	39.69	39.73 (0.10)	39.47 (-0.55)	39.47 (-0.55)	39.15 (-1.36)
	*Obese*	31.19	31.15 (-0.12)	31.00 (-0.60)	31.00 (-0.60)	30.75 (-1.39)
SSB Q3 (n = 711)	*Normal*	28.04	29.01 (3.47)	29.51 (5.25)	29.48 (5.15)	30.90 (10.22)
	*Overweight*	35.53	35.25 (-0.78)	35.76 (0.63)	36.02 (1.37)	35.27 (-0.73)
	*Obese*	36.43	35.74 (-1.90)	34.73 (-4.66)	34.50 (-5.30)	33.83 (-7.15)
SSB Q4 (n = 621)	*Normal*	33.07	34.81 (5.25)	39.22 (18.60)	39.54 (19.56)	44.84 (35.58)
	*Overweight*	29.99	30.96 (3.23)	30.59 (1.98)	30.67 (2.26)	28.12 (-6.23)
	*Obese*	36.94	34.23 (-7.33)	30.19 (-18.26)	29.79 (-19.35)	27.04 (-26.80)

1 Sugar sweetened beverages (SSB) includes: carbonated beverages, *aguas frescas* and juices (industrialized).

2 BMI cut-off points: Normal < 25, Overweight 25–29.99, Obese ≥ 30 kg/m^2^.

3 Population expansion: Total: 63,151,429, Q1: 14,658,319, Q2: 16,469,474, Q3: 16,233,311, Q4: 15,790,324.

4 Taxed sugar sweetened beverages quartile categories (ml/person/day): Q1: ≤33.94, Q2: 33.94–129.64, Q3: 129.64–402.86, Q4: ≥ 402.86.

Table 5 presents projections of diagnosed diabetes prevalence and averted cases by 2030 and 2050 under the 10% and 20% tax for average and peak monthly effect scenarios. The ranges capture the variability associated with the three future diabetes incidence assumptions. By 2050, the prevalence of diagnosed diabetes in adults (20 or older) is projected to be 13.6% to 22.5%. Under the average effect of the 10% tax, diabetes prevalence would decrease in 2050 to 13.4% to 22.2% (13.3% to 22.2% assuming the peak monthly effect). These changes would translate into 86K to 134K averted cases by 2030 under average and 161K to 251K averted cases under peak monthly effect. These would increase to 225K to 324K averted cases by 2050 under the average effect, and 421K to 605K under the peak monthly effect. The hypothetical 20% tax would produce further reductions of diabetes prevalence, as it roughly duplicates the number of prevented cases. S3 Fig in S1 File shows projected annual diagnosed diabetes prevalence and the annual number of averted cases for all tax scenarios from 2015–2050 under the intermediate incidence assumption.

**Table 5:** Expected prevalence, prevalence change and averted cases of diagnosed type-2 diabetes in the adult Mexican population from 2015–2050.

	By year	No tax	10% tax		20% tax	
			*Average*	*Peak Monthly*	*Average*	*Peak Monthly*
**Prevalence**	2030	13.0 (12.1–18.5%)	12.9 (11.9–18.3%)	12.8 (11.9–18.2%)	12.8 (11.9–18.2%)	12.6 (11.7–18.0%)
	2050	14.9 (13.6–22.5%)	14.7 (13.4–22.2%)	14.6 (13.3–22.2%)	14.6 (13.3–22.2%)	14.3 (13.0–23.6%)
**Prevented cases** **(thousands)**	2030	-	92 (86–134)	173 (161–251)	184 (171–267)	341 (317–497)
	2050	-	240 (225–324)	448 (421–605)	476 (448–645)	886 (833–1,202)

## Discussion

The present study estimated the expected changes in BMI and the prevalence of overweight, obesity and diabetes in Mexican adults after the implementation of the 1-peso-per-litre tax to SSB in Mexico and under a hypothetical 20% tax scenario. Considering the 6.1% reduction in SSB purchases observed in 2014, after 10 years of implementation, the tax is expected to reduce an average 0.15 kg/m^2^ per person; the 20% tax would induce an average reduction of 0.31 kg/m^2^ per person. These BMI reductions would translate into sizable changes in the prevalence of obesity: 2.54% with respect to baseline for the 10% tax, and 6.85% for the 20% scenario. As for diabetes, we project that the 10% tax would prevent 86K-134K cases by year 2030, compared to 171K-267K cases with a 20% tax.

To our knowledge, this is the first projection of the potential health impact of a nation-wide SSB tax based on observed changes in SSB purchases after tax implementation. While the 0.15 kg/m^2^ reduction achieved with the tax could seem small from an individual level perspective, achieving this reduction at the population level with a single intervention is relevant, as it translates into a 2.54% reduction in the obesity prevalence 10 years after the tax; further reductions are expected among heavy consumers (7.33% lower prevalence of obesity). Our simulations also shed light on two important aspects of the tax. First, SSB consumption is concentrated in the younger age groups who benefit the most from the tax in terms of caloric and BMI reductions. This trend was observed for both men and women. BMI reductions earlier in life are likely to offer important health benefits throughout the life course, such as lower incidence of diabetes and cardiovascular disease.[43–45] Also, as S1 File Tables S1 to S3 show, larger reductions in BMI were observed at lower SES levels for men and in most scenarios for women. The finding that larger declines in BMI were observed in the low SES is important. It means that the benefits derived from the reduction in purchases due to the tax favours the poorer households in terms of lower risks of obesity and chronic diseases in the medium and long run, which translates into savings due to reductions in medical attention and gains in productivity.

Sugar-sweetened beverages are major drivers of diabetes in Mexico.[46] According to our projections under an intermediate incidence scenario 14.9% of the population would be diagnosed with diabetes by 2050; the average effect of the 10% tax could avert 240K cases (1.3% change in prevalence). However, if the peak monthly effect of 2014 was to be maintained, averted cases could amount to 448K (2.0% change in prevalence). According to our estimates the 20% tax could duplicate the benefits of the current tax. This finding goes in the same direction as simulation studies from low and middle-income countries, such as India (-1.6% incidence in 10 years under a hypothetical 20% tax) and South Africa (-4% prevalence over 20 years with a hypothetical 20% tax).[19,23] Similar findings have been reported in Australia and the United States of America.[24,47] A recent study by Sanchez-Romero, et al., estimated the expected effect of the SSB tax in Mexico on cardiovascular disease and diabetes.[25] Assuming that the tax would translate in a 10% reduction in consumption with 39% compensation, from 2013 to 2022 they estimated 189K fewer diabetes cases, 20K fewer stroke and myocardial infarction cases, and 19K fewer deaths. The only common outcome to our study is diabetes but scenarios have substantial differences. In their no substitution scenario, Sanchez-Romero, et al., projected 265K (220-300K) fewer diabetes cases from 2013 to 2022 (26.5/year in average); in contrast, under the 12% reduction scenario, from 2015 to 2030 we project 173K (160-250K) prevented diagnosed diabetes cases. Assuming that in 2012 35% diabetes cases were undiagnosed that would roughly take us to 233K in 2030 (14.6K/year). Thus, they estimate twice as much of a reduction. However, these projections are not directly comparable, because of important differences in model assumptions. In particular, Sanchez-Romero, et al., modeled the incidence of total diabetes (diagnosed and undiagnosed) based on a risk model developed for the US population (the Framingham diabetes risk model).[25] In contrast, our projections are based on age-specific incidence of diagnosed diabetes estimated directly from the Mexican population.[40] Sources to estimate total diabetes projections based in Mexican data are not yet available,[40] future studies will need to refine these estimates once data becomes available. Despite differences, both models suggest that the tax could have a considerable impact to reduce the projected burden of diabetes in Mexico. As the prevalence of diabetes increases in Mexico strong calls to find structural solutions to the diabetes epidemic have been made.[48] Our findings point towards the importance of reducing SSB consumption as a key part of an integral approach to diabetes prevention.

Mexico provides a particularly suitable environment to evaluate the potential impact of a SSB tax. SSB consumption and the prevalence of overweight, obesity and diabetes are very high; thus, reductions associated to a decrease in SSB consumption should be more evident than in countries with lower SSB consumption. Our expected caloric decrease is larger than what models in countries such as the UK, India and Ireland have found, and similar to estimates from the US. A 10% tax would induce a 2.1 kcal/person/day decrease in Ireland, 4.0 kcal/person/day in UK, 4.3 kcal/person/day in India, and 18.0 kcal/person/day in the US;[19–22] in comparison, we estimated a decrease of 8.38 kcal/person/day in Mexico after the 10% tax using the average decrease observed in 2014. Differences in caloric reduction depend on multiple factors, such as SSB baseline consumption, SSB caloric content, price elasticity and caloric substitution based on availability of substitutes in each country.

The 1-peso-per-litre tax is an excise tax, only affecting industrialized drinks with sugar and with no effect over homemade beverages. According to our data, Mexicans consume 189 kcals/day from SSB, yet, our simulation is based only on industrialized drinks, which represent 66.4% of the SSB caloric intake. This is a major difference compared to other studies, which have modelled caloric and weight reductions associated to all SSB. Thus, average caloric intake from SSB in the UK has been estimated on 29.4 kcal/person/day, India 46 kcal/person/day, and 153 kcal/person/day in the US.[20–22] The definition of SSB presented in previous studies assume a tax that affects both homemade and industrialized SSB, which would require a tax to sugar, rather than to SSB. However, such tax would be difficult to implement, as it would simultaneously affect all industries and products that use sugar. This distinction is irrelevant for countries where homemade SSB are rare, but in countries like Mexico the exclusion of homemade SSB is important to avoid overestimation of the tax impact.

Our study has important limitations. Our estimate of the tax effect is based on previous work based on purchase surveys.[18] We acknowledge that survey data may underestimate SSB purchases or consumption. However, a recent paper using aggregate sales data, collected by the Mexican National Institute of Statistics and Geography, shows a 6.2% reduction of SSB sales in 2014,[49] a very similar result compared to the parameter used to estimate changes in consumption after the SSB tax was implemented of 6%. Also, our approach to estimate BMI change assumes substitution towards non-caloric alternatives. Yet, substitutions for other caloric beverages are unlikely for a number of reasons. Firstly, the SSB tax implemented in Mexico includes all beverages with added sugar, which reduces potential substitutions for caloric beverages. Secondly, two studies have found increases in untaxed beverages (beverages with no added sugar) after the tax was implemented. The first study used a panel of households and showed a 4% increase in purchases of untaxed beverages in 2014 (mainly bottled water).[18] The second study used aggregate sales data and found a 5.2% increase in sales of plain water manufactured by the beverage industry.[49] These findings suggest that after the tax the population substituted for untaxed beverages, mainly plain water. However, we also conducted a sensitivity analysis in which we used estimates of the direct effect of SSB consumption changes in body weight (i.e., not through calorie mediation via the Hall Model) from studies that account for regular dietary substitution adjusting for simultaneous changes in other nutritional variables and from experimental studies that modified SSB consumption while allowing free-feeding for other foods. For the sensitivity analysis we used comparative risk assessment models, [50] rather than Hall and Chow’s model (details of the implementation of the sensitivity analysis available in S2 File Section 3). Using baseline weight as the comparison, we calculated the limits of the expected annual weight change after the tax using the average experimental and observational meta-analytical effects (0.85 kg/m^2^ per serving/day experimental, 0.22 kg/m^2^ per serving/day observational) estimated by *Malik*, *et al*.[51] Given the large heterogeneity observed in the meta-analysis of observational studies we also included the effects of three specific studies: the lowest (0.11 kg/m^2^ per serving/day for Mozaffarian),[52] middle (1.09 kg/m^2^ per serving/day for Chen)[53] and highest (2.12 kg/m^2^ per serving/day for Barone).[54] S4 Fig in S1 File presents expected BMI changes 10 years after the tax under different BMI change per SSB serving scenarios, assuming the average 6.1% decrease; all scenarios produce a decrease in BMI, and our estimate falls within the range of values produced using observational coefficients for BMI change and close to those generated with experimental coefficients. We estimated weight change using the Mexican population who participated in the ENSANUT 2012. Some participants had chronic diseases that could affect weight change over time, such as diabetes. Modeling disease-specific weight change rates is complex, and no data is available for the Mexican population; therefore, we had to assume that weight change was similar and properly captured by Hall’s model across the population. Chronic diseases tend to produce weight decreases, so our average estimate for weight after 10 years of the tax could be an underestimate of the expected population weight. This, however, will not necessarily affect the estimated weight impact of the tax, assuming that the benefits of reducing caloric intake from SSB are the same independently the health status of the people. This issue does not affect diabetes projections, since models are based on people with no diabetes at baseline.

Our diabetes model is restricted to diagnosed diabetes, underestimating the potential tax effect on total diabetes. We did not consider total diabetes (undiagnosed and diagnosed cases), because there is no data available for Mexico, except for the ENSANUT-2006. Our model only considered adults, and no evaluation on children was conducted. Children are heavily targeted by the SSB industry and consume important quantities of SSB. Future efforts must be conducted to estimate the effect of SSB taxes on children, as they are likely to benefit the most from this policy.

Our study also has several strengths. This is the first estimate using the observed effect of a nationwide excise tax to SSB; as such, it provides data and evidence that is closer to what other countries may experience if similar taxes are enforced. We provided estimates for both the average effect observed during 2014 (6.1% decrease) and the peak monthly effect recorded during that year (11.9% decrease); previous estimates of the elasticity of SSB suggested a 10% increase was expected to produce a 12.1% decrease in consumption, which is consistent with the observed value in December 2014. It is difficult to establish how long will it take for the tax to reach its stable effect. While we decided to present the more conservative 6.1% decrease, the 11.9% scenario is also likely. Our weight change analysis relied in nationally representative individual-level data, which allowed us to provide stratum-specific effects to evaluate potential differences in the expected effect of the tax. We used Chow and Hall’s dynamic weight-change model, which provides a closer resemblance to the physiological weight change process and has shown to produce valid estimates;[35–38] also, it has been widely used in the literature, among other things to model the impact of SSB taxes on weight change [39] allowing us to compare to previous estimates.[22,39] A common concern of implementing a SSB tax is that it could lead to larger and unhealthy BMI reductions among people in the lowest SES levels; by using SES-specific elasticities and data we showed such concerns are unsubstantiated as both high and low SES people experienced similar BMI reductions. We used a diabetes model that is based on the Mexican trajectories of diagnosed diabetes, providing locally grounded information of future trends and prevented cases due to the tax.

## Conclusion

An expert panel in Mexico recently recommended increasing the tax to 20%.[55] This recommendation has found echo in the Senate and House of Representatives of Mexico and has been put to discussion every year since 2015. The proposal has not yet been accepted. In 2016 Mexico launched an epidemiological emergency call for obesity and diabetes, to increase the actions leading to their reduction. Our study suggests an important opportunity to further reduce body weight and diabetes, as doubling the tax may produce health benefits that in some instances more than double those of the 10% tax. Within this context, and as evidence of the cost-effectiveness of taxes to SSB increases,[47] taxes to highly caloric foods could become a critical tool to reduce obesity and diabetes in the country.

SSB taxes may take many forms,[56] yet the Mexican tax will likely be reproduced in other countries. The implementation of the tax is expected to produce important population weight and diabetes risk reductions. Larger BMI reductions were observed in low SES and younger groups, suggesting larger health gains among the poor and the youth. Diabetes cases could also be considerably reduced. The Mexican experience points towards SSB taxes being useful to reduce consumption and produce sizable gains in weight and diabetes reduction.

## Supporting information

S1 File Additional results. Main model diagrams, and additional results on Body Mass Index and Diabetes.
(PDF)

S2 File Methodology. Additional information on the diabetes and weight change models as well as the sensitivity analysis.
(PDF)
