# Correction: Expected population weight and diabetes impact of the 1-peso-per-litre tax to sugar sweetened beverages in Mexico

**DOI:** 10.1371/journal.pone.0191383

**Published:** 2018-01-11

**Authors:** Tonatiuh Barrientos-Gutierrez, Rodrigo Zepeda-Tello, Eliane R. Rodrigues, M. Arantxa Colchero, Rosalba Rojas-Martínez, Eduardo Lazcano-Ponce, Mauricio Hernández-Ávila, Juan Rivera-Dommarco, Rafael Meza

The fourth author's name is spelled incorrectly. The correct name is: M. Arantxa Colchero. The correct citation is: Barrientos-Gutierrez T, Zepeda-Tello R, Rodrigues ER, Colchero MA, Rojas-Martínez R, Lazcano-Ponce E, et al. (2017) Expected population weight and diabetes impact of the 1-peso-per-litre tax to sugar sweetened beverages in Mexico. PLoS ONE12(5): e0176336. https://doi.org/10.1371/journal.pone.0176336

S1 File is incorrect. The corrected version can be viewed below.

## Supporting information

S1 File Additional Results. Main model diagrams, and additional results on Body Mass Index and Diabetes.
(PDF)
