# Supplementary material for: Expected population weight and diabetes impact of the 1-peso-per-litre tax to sugar sweetened beverages in Mexico
Source: PLoS One. 2017 May 17;12(5):e0176336. doi: 10.1371/journal.pone.0176336 (PMC5435164; doi:10.1371/journal.pone.0176336)
Supplement: S1 File — Main model diagrams, and additional results on Body Mass Index and Diabetes. (PDF) [file pone.0176336.s001.pdf]

Appendix

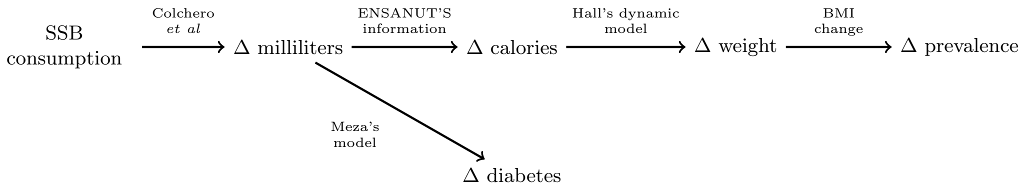

Figure S1: Diagram of the model integration process to project the tax impact on diabetes prevalence.

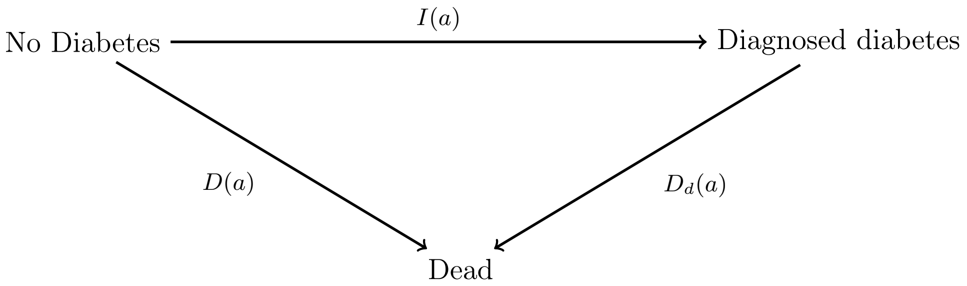

Figure S2: Diabetes Natural History Model. Individuals are broken in 101 age categories (ages 0 to 100) and 2 disease states (without diabetes and with diagnosed diabetes). Incidence of diabetes ($I(a)$) varies by age and gender. Individuals with diabetes die at a higher age and gender-specific rate ($D_d(a)$) than individuals without diabetes ($D(a)$). Model was parametrized using the estimated Mexico Diabetes incidence and prevalence from ENSANUT. Mortality rates come from CONAPO and future births are based on census projections.

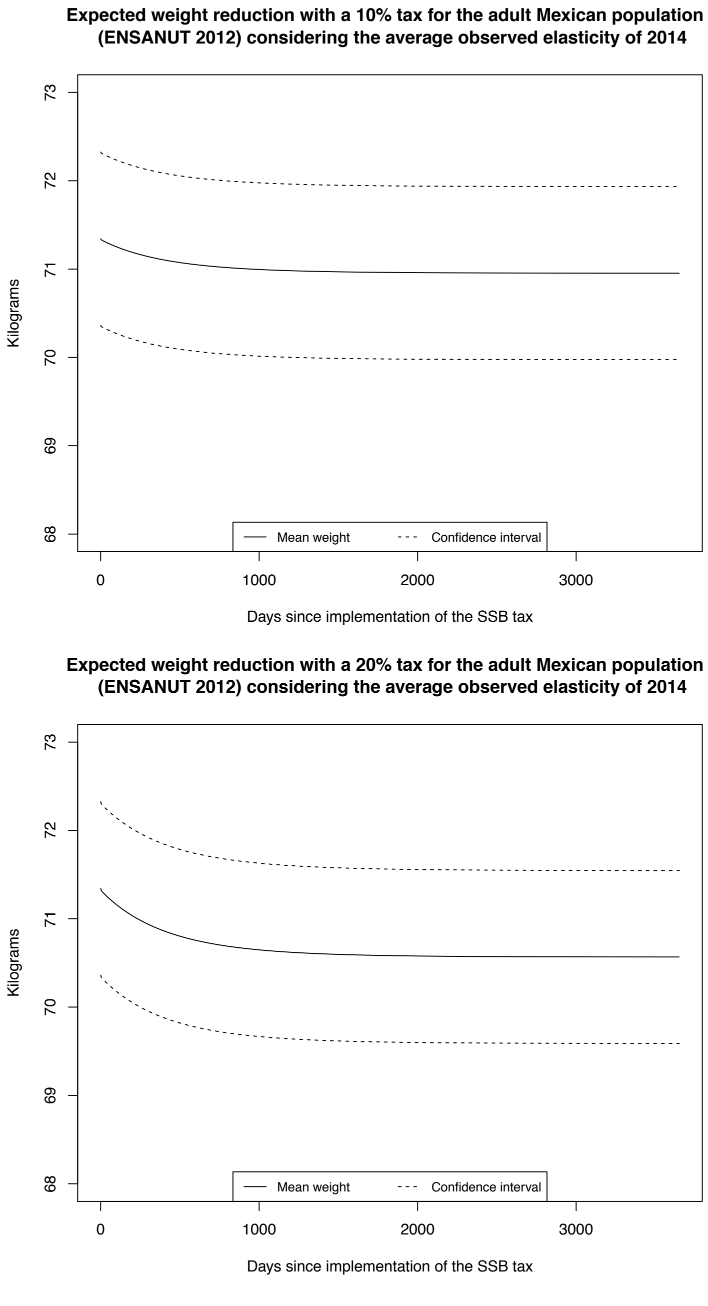

Figure S3: Population level expected weight reduction for a 10 and 20% tax considering the average change for 2014

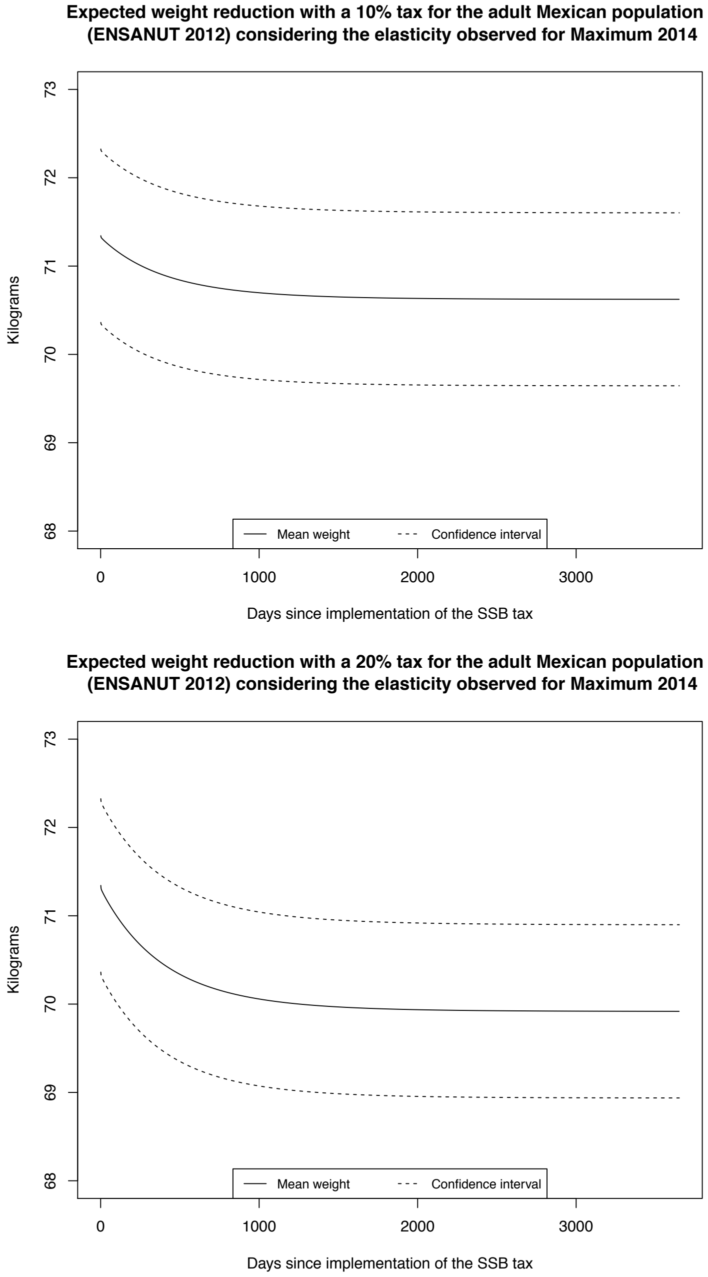

Figure S4: Population level expected weight reduction for a 10 and 20% tax considering the peak monthly tax effect observed in 2014

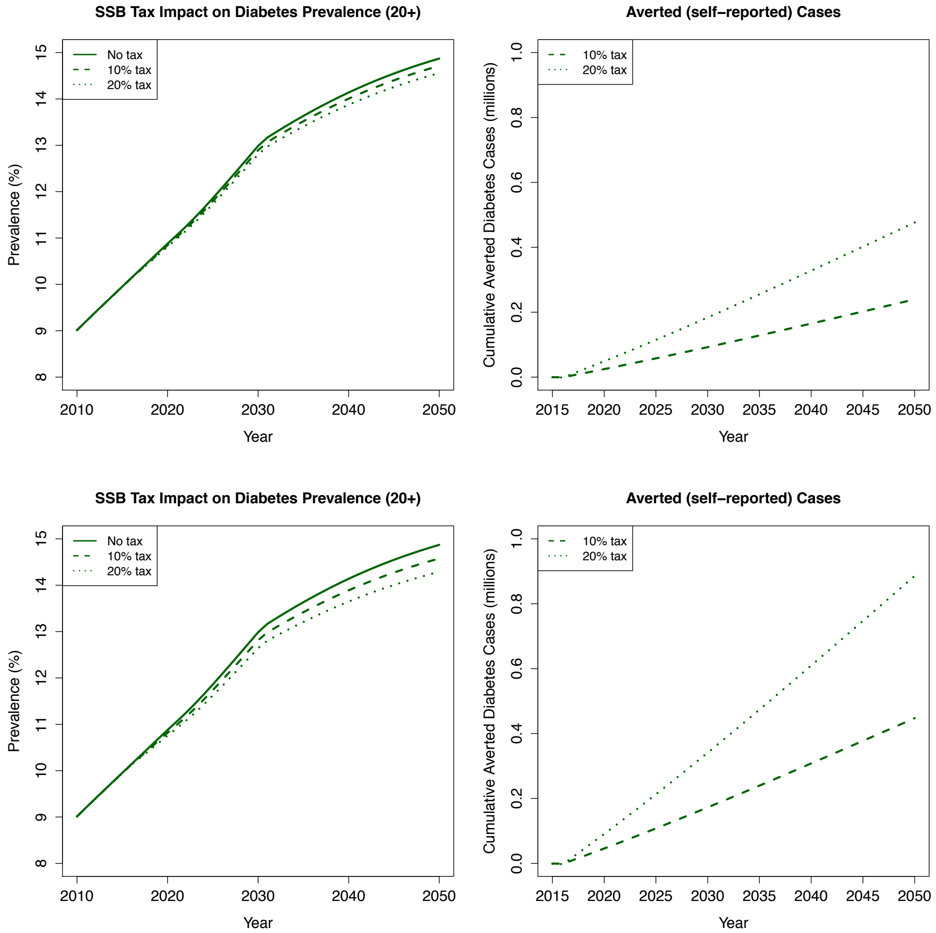

Figure S5: Projection of diabetes (self-reported) prevalence and cumulative averted diabetes cases under three tax scenarios from 2010-2015. Top, projections assuming the average observed change of 2014. Bottom, projections assuming the peak monthly tax effect of 2014.

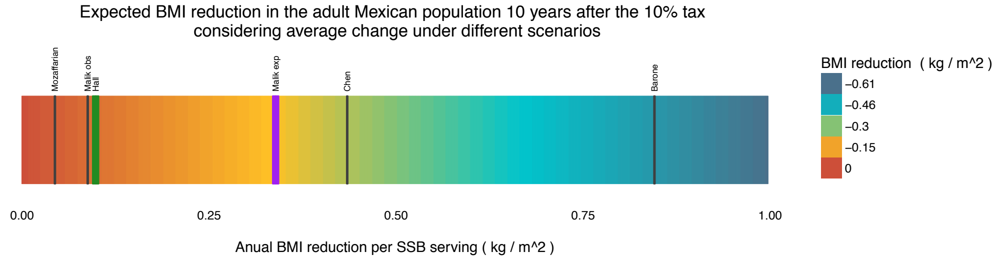

Figure S6: Expected BMI change under CRA.

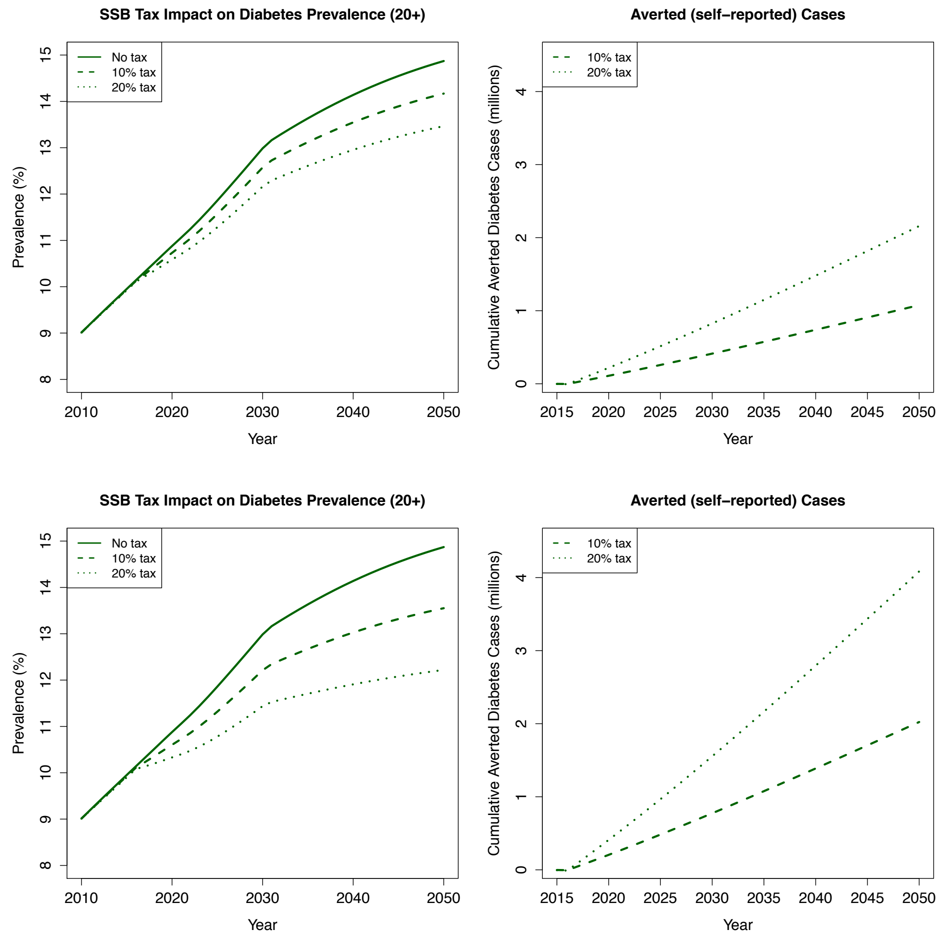

Figure S7: Projection of diabetes (self-reported) prevalence and cumulative averted diabetes cases under three tax scenarios from 2010-2015 based on weight change. Top, projections assuming the average observed change of 2014. Bottom, projections assuming the peak monthly tax effect of 2014.

Table S1: Mean body mass index (BMI) change (kg/m^2) in the adult Mexican population (ENSANUT 2012) before and after the implementation of a SSB[a] tax at 10, and 20% assuming the average and peak monthly observed changes of 2014 (n=2,735)[b].

		10% tax				20% tax			
	Baseline	Year 1		Year 10		Year 1		Year 10	
		Average effect	Peak monthly effect	Average effect	Peak monthly effect	Average effect	Peak monthly effect	Average effect	Peak monthly effect
**Total**	28.40	28.31	28.23	28.25	28.11	28.22	28.06	28.09	27.83
**Socioeconomic status**									
Low	27.75	27.63	27.53	27.56	27.38	27.51	27.30	27.36	27.01
Medium	28.74	28.66	28.55	28.60	28.41	28.58	28.36	28.46	28.09
High	28.72	28.65	28.63	28.59	28.56	28.58	28.54	28.47	28.41
**Age (years)**									
20 - < 40	27.83	27.72	27.63	27.65	27.50	27.61	27.42	27.47	27.17
40 - < 60	29.59	29.51	29.44	29.45	29.31	29.43	29.28	29.30	29.04
≥ 60	27.66	27.60	27.56	27.56	27.48	27.54	27.45	27.45	27.29
**Quartiles of SSB consumption**[c]									
Q1	28.34	28.34	28.34	28.34	28.34	28.34	28.34	28.34	28.34
Q2	28.24	28.22	28.20	28.20	28.17	28.20	28.16	28.16	28.10
Q3	28.64	28.57	28.51	28.52	28.42	28.50	28.39	28.40	28.20
Q4	28.37	28.10	27.87	27.92	27.53	27.84	27.37	27.47	26.71

[a] *Taxed sugar sweetened beverages (SSB) include: soft drinks, aguas frescas and juices (industrialized)*
[b] *Expands to 63,151,429 individuals.*
[c] *Taxed sugar sweetened beverages quartile categories (ml/day): Q1: ≤33.94, Q2: 33.94-129.64, Q3: 129.64-402.86, Q4: ≥402.86*

Table S2: Mean body mass index (BMI) change (kg/m^2) in the male adult Mexican population (ENSANUT 2012) before and after the implementation of a SSB[a] tax at 10, and 20% considering the average and peak monthly observed effects of 2014 (n=1,080)[b].

		10% tax				20% tax			
	Baseline	Year 1		Year 10		Year 1		Year 10	
		Average effect	Peak monthly effect	Average effect	Peak monthly effect	Average effect	Peak monthly effect	Average effect	Peak monthly effect
**Total**	27.55	27.44	27.34	27.37	27.22	27.33	27.13	27.19	26.89
**Socioeconomic status**									
Low	26.99	26.84	26.69	26.74	26.51	26.68	26.38	26.49	26.05
Medium	27.43	27.33	27.21	27.28	27.07	27.24	26.98	27.12	26.72
High	28.23	28.15	28.13	28.09	28.06	28.07	28.03	27.96	27.90
**Age (years)**									
20-< 40	27.40	27.26	27.14	27.18	27.00	27.12	26.88	26.97	26.61
40 - < 60	28.16	28.06	27.96	27.99	27.83	27.96	27.76	27.82	27.51
≥ 60	26.86	26.79	26.75	26.75	26.67	26.72	26.63	26.63	26.48
**Quartiles of SSB consumption**[c]									
Q1	27.51	27.51	27.51	27.51	27.51	27.51	27.51	27.51	27.51
Q2	27.13	27.10	27.09	27.09	27.06	27.08	27.05	27.06	27.00
Q3	27.68	27.62	27.56	27.58	27.48	27.55	27.44	27.47	27.29
Q4	27.75	27.48	27.23	27.31	26.92	27.20	26.71	26.87	26.12

[a] *Taxed sugar sweetened beverages (SSB) include: soft drinks, aguas frescas and juices (industrialized)*
[b] *Expands to 28,333,441 individuals*
[c] *Taxed sugar sweetened beverages quartile categories (ml/day): Q1: ≤33.94, Q2: 33.94-129.64, Q3: 129.64-402.86, Q4: ≥402.86*

Table S3: Mean body mass index (BMI) change (kg/m^2) in the female adult Mexican population (ENSANUT 2012) before and after the implementation of a SSB[a] tax at 10, and 20% assuming the average and peak monthly observed effects of 2014 (n=1,655)[b].

		10% tax				20% tax			
	**Baseline**	**Year 1**		**Year 10**		**Year 1**		**Year 10**	
		Average effect	Peak monthly effect	Average effect	Peak monthly effect	Average effect	Peak monthly effect	Average effect	Peak monthly effect
**Total**	29.09	29.02	28.96	28.96	28.85	28.95	28.82	28.83	28.60
**Socioeconomic status**									
Low	28.38	28.30	28.22	28.24	28.10	28.22	28.06	28.09	27.82
Medium	29.77	29.70	29.60	29.63	29.45	29.62	29.43	29.50	29.15
High	29.12	29.06	29.04	29.00	28.98	28.99	28.96	28.88	28.83
**Age (years)**									
20- 40	28.14	28.06	27.99	27.99	27.87	27.97	27.83	27.84	27.60
40 - 60	30.77	30.70	30.64	30.64	30.53	30.64	30.52	30.52	30.29
$\geq$ *60*	28.48	28.42	28.38	28.38	28.29	28.37	28.27	28.28	28.11
**Quartiles of SSB consumption**[c]									
Q1	28.76	28.76	28.76	28.76	28.76	28.76	28.76	28.76	28.76
Q2	28.96	28.94	28.92	28.92	28.88	28.91	28.87	28.87	28.80
Q3	29.57	29.50	29.44	29.45	29.33	29.43	29.31	29.32	29.09
Q4	29.19	28.93	28.71	28.72	28.33	28.68	28.24	28.27	27.50

[a] *Taxed sugar sweetened beverages (SSB) include: soft drinks, aguas frescas and juices (industrialized)*

[b] *Expands to 34,817,989 individuals*

[c] *Taxed sugar sweetened beverages quartile categories (ml/day): Q1: $\leq$33.94, Q2: 33.94-129.64, Q3: 129.64-402.86, Q4: $\geq$402.86*

Table S4: Expected prevalence, prevalence change and averted cases of diagnosed type 2 diabetes in the adult Mexican population from 2015-2050 based on weight change.

	By year	No tax	10% tax		20% tax	
			Average	Peak Monthly	Average	Peak Monthly
**Prevalence**	2030	12.1-18.5%	10.8-16.4%	10.4-15.7%	10.4-15.6%	9.5-14.3%
	2050	13.6-22.5%	11.9-19.5%	11.2-18.4%	11.1-18.2%	9.7-16.0%
**Prevalence Change**[a]	2030	-	10.1-13.8%	13.5-17.1%	13.9-17.5%	20.7-24.0%
	2050	-	12.3-16.8%	17.4-21.7%	18.1-22.3%	28.4-32.1%
**Prevented Cases**[b]	2030	-	383-599	718-1,128	765-1,204	1,440-2,285
	2050	-	1,010-1,456	1,897-2,752	2,023-2,941	3,822-5,630

[a]Relative with respect to expected prevalence.
[b]Cumulative (in thousands).
