# Supplementary material for: Expected population weight and diabetes impact of the 1-peso-per-litre tax to sugar sweetened beverages in Mexico
Source: PLoS One. 2017 May 17;12(5):e0176336. doi: 10.1371/journal.pone.0176336 (PMC5435164; doi:10.1371/journal.pone.0176336)
Supplement: S2 File — Additional information on the diabetes and weight change models as well as the sensitivity analysis. (PDF) [file pone.0176336.s002.pdf]

Appendix 2

1 Weight-change model

1.1 Mexican data

As specified in the main article we used data from the National Health and Nutrition Survey (ENSANUT) which can be found in [1]. For that purpose we merged the adult, home and food frequency questionnaires using the variables `folio` and `intp` which identify respectively the home and the individual inside the home. The resulting dataset called `Adult_complete_ENSANUT_dataset.rda` can be found at the Open Science Framework for replication purposes `https://osf.io/khkwp/#`. Table 1 details the variables in the dataset.

Name	Variable
`foliointp`	Unique identifier for each individual in ENSANUT's dataset.
`folio_c`	Unique identifier for each home in ENSANUT's dataset.
`intp`	Identifier of the individual inside the Home.
`mlsoda`	Daily consumption of taxed soda (ml).
`mlaguasfrescas`	Daily consumption of taxed *aguas frescas* (ml).
`mljuice`	Daily consumption of taxed juice (ml).
`mlmilk`	Daily consumption of milk (ml).
`mlssb`	Daily consumption of SSB (`mlssb` = `mlsoda` + `mlaguasfrescas` + `mljuice`).
`age_yrs`	Age (yrs) averaged throughout the dates in which the questionnaires were applied.
`pondef`	Complex survey weight.
`code_upm`	Indentifier of Primary Sampling Units.
`est_var`	Strata for the estimation of variances accounting for survey design.
`tertile`	Socioeconomic tertile estimated via the weighted sample.
`sex`	Sex of the individual: coded as 1 if male; 2 if female.
`weight`	Average weight (kg) of the individual (2 measurements).
`height`	Average height (m) of the individual (2 measurements).
`agecat`	Age category. Levels: "20 to 40", "40 to 60", "Over 60".
`bmi`	Body Mass Index (numeric).
`bmi_oms`	Body Mass Index category according to WHO.

Table 1: Variables in `Adult_complete_ENSANUT_dataset.rda`

To account for the complex survey design we used the `R` package `survey` [2] with the following design:

```
svystr <- svydesign(id = ~foliointp , strata = ~est_var,
                    weights = ~pondef , PSU = ~code_upm, data = Adults)
options(survey.lonely.psu = "adjust")
```

All population-level estimations were done considering that design.

1.2 Caloric and sodium intake change

According to ENSANUT's documentation, each milliliter of taxed soda is equivalent to 0.398 kcals, taxed aguas frescas to 0.2 kcals, and taxed juices to 0.3944 kcals. Using the information on consumption from the variables `mlsoda`, `mljuice` and `mlaguasfrescas` we obtained information of caloric intake attributable to

SSB. For sodium consumption attributable to SSB the same process was followed with: 0.1731, 0.1545, and 0.0884 mg of sodium associated to taxed sodas, aguas frescas and juices respectively.

In order to estimate consumption change we classified individuals according to their socioeconomic `tertile` and applied the formula for each individual i:

$$\Delta \text{cal}^{(i)} = \text{Tertile specific elasticity} \times \text{Consumption in kcals}^{(i)}. \tag{1}$$

Sodium change was quantified in an equivalent fashion into the variable $\Delta \text{Na}_{diet}^{(i)}$

1.3 Weight model

The weight change model [3] describes weight (in kilograms) for an individual i by the following time-dependent function:

$$BW^{(i)}(t) = ECF^{(i)}(t) + 3.7(G^{(i)}(t) - G_{init}) + F^{(i)}(t) + L^{(i)}(t), \tag{2}$$

where $F^{(i)}(t), L^{(i)}(t), ECF^{(i)}(t), G^{(i)}(t)$ represent the masses of fat and lean tissues, extracellular fluids and glycogen of individual i. G_{init} represents the stable initial glycogen mass of 0.5 kg.

The extracellular fluid mass $ECF^{(i)}(t)$ is the solution to the differential equation:

$$\frac{dECF^{(i)}}{dt} = \frac{1}{Na}\Big(\Delta Na_{diet}^{(i)} + \xi_{Na}(ECF^{(i)} - ECF_{init}^{(i)}) - \xi_{CI}(1 - CI^{(i)}/CI_b^{(i)})\Big). \tag{3}$$

which can be represented analytically as:

$$\begin{aligned} ECF^{(i)}(t) = \frac{1}{CI_b \xi_{diet}} e^{-\frac{t\xi_{diet}}{Na}} \Big(& CI^{(i)}\xi_{CI} e^{\frac{t\xi_{diet}}{Na}} - CI^{(i)}\xi_{CI} + CI_b^{(i)} ECF_{init}\xi_{diet} e^{\frac{t\xi_{diet}}{Na}} \\ & + CI_b^{(i)}\Delta Na_{diet} e^{\frac{t\xi_{diet}}{Na}} - CI_b^{(i)}\Delta Na_{diet} - CI_b^{(i)}\xi_{CI} e^{\frac{t\xi_{diet}}{Na}} + CI_b^{(i)}\xi_{CI}\Big), \end{aligned} \tag{4}$$

where $Na = 3.22$ mg/ml, $\xi_{Na} = 3000$ mg/L/d, and $\xi_{CI} = 4000$ mg/d, are phisiological constants further described in the appendix of [3]. $\Delta Na_{diet}^{(i)}$ represents the change in sodium (mg/d) for individual i according to (1). $CI_b^{(i)}$ the carbohidratic intake at baseline (assumed to be 1/2 of energy intake estimated in (1)) and $CI^{(i)}$ the carbohidratic intake after the consumption reduction (assumed to be 1/2 of energy intake after reduction $EI^{(i)}$ which we explain in (14)).

Glycogen mass $G(t)$ is given by the solution to the differential equation:

$$\frac{dG^{(i)}}{dt} = \frac{1}{\rho_G}\Big(CI^{(i)} - k_G^{(i)}(G^{(i)})^2\Big), \tag{5}$$

where $\rho_G = 4206.501$ kcals/kg ($17.6 MJ/kg$), and $k_G^{(i)} = CI_b^{(i)}/G_{init}^2$ represent biological invariates.

Finally, $F^{(i)}(t)$ and $L^{(i)}(t)$ represent the solutions to the following system of nonlinear ordinary differential equations:

$$\begin{aligned} \frac{dF^{(i)}}{dt} &= \frac{(1 - p^{(i)})}{\rho_F}\Big(EI^{(i)} - EE^{(i)} - \rho_G\frac{dG^{(i)}}{dt}\Big), \\ \frac{dL^{(i)}}{dt} &= \frac{p^{(i)}}{\rho_L}\Big(EI^{(i)} - EE^{(i)} - \rho_G\frac{dG^{(i)}}{dt}\Big). \end{aligned} \tag{6}$$

with $\rho_F = 9440.727$ kcals/kg (39.5 MJ/kg), $\rho_L = 1816.444$ kcals/kg (7.6 MJ/kg) are constants, and $p^{(i)} \equiv p^{(i)}(t) = C/\big(C + F^{(i)}(t)\big)$ with $C = 10.4 \cdot \rho_L \rho_F^{-1}$ is a function of the fat mass.

Total energy expenditure rate $EE^{(i)}$ is given by:

$$EE^{(i)} = K^{(i)} + \gamma_F F^{(i)} + \gamma_L L^{(i)} + \delta^{(i)} BW^{(i)} + TEF^{(i)} + AT^{(i)} + \eta_L\frac{dL^{(i)}}{dt} + \eta_F\frac{dF^{(i)}}{dt} \tag{7}$$

with $\gamma_F = 3.107075$ kcals/kg/d (13 kJ/kg/d), $\gamma_L = 21.98853$ kcals/kg/d (92 kJ/kg/d), $\eta_F = 179.2543$ kcals/kg (750 kJ/d), $\eta_L = 229.4455$ kcals/kg (960 kJ/kg) are physiological constants.

$K^{(i)}$ is determined by the initial energy balance condition:

$$K^{(i)} = 0.76RMR^{(i)} \cdot PAL - \gamma_L L^{(i)}(0) - \gamma_F F^{(i)}(0) - \delta^{(i)} BW^{(i)}(0) \tag{8}$$

with $RMR^{(i)}$ the initial resting metabolic rate (as estimated by (13)), PAL the physical activity level (assumed $PAL = 1.5$). The constant $\delta^{(i)}$ is given by:

$$\delta^{(i)} = \frac{(1 - \beta_{TEF}) \cdot PAL - 1}{BW^{(i)}(0)} RMR^{(i)} \tag{9}$$

with $\beta_{TEF} = 0.1$.

The thermal effect of feeding is quantified by:

$$TEF^{(i)} = \beta_{TEF} \Delta EI^{(i)} \tag{10}$$

with $\Delta EI^{(i)}$ the change on energy intake.

Finally, adaptative thermogenesis is given by the function:

$$AT^{(i)}(t) = \beta_{AT} \Delta EI^{(i)} + e^{-t/\tau_{AT}} (-\beta_{AT} \Delta EI^{(i)} + AT_{init}^{(i)}) \tag{11}$$

which is the solution to:

$$\frac{dAT^{(i)}}{dt} = \beta_{AT} \Delta EI^{(i)} - AT^{(i)} \tag{12}$$

with $AT_{init}^{(i)} = \beta_{AT} \cdot PAL \cdot RMR^{(i)}$.

We remark that for each individual Resting Metabolic Rate $RMR^{(i)}$ was obtained using the formula by [4]:

$$RMR^{(i)} = \begin{cases} 9.99 \times \texttt{weight} + 625 \times \texttt{height} - 4.92 \times \texttt{age\_yrs} + 5 & \text{if } \texttt{sex} = 1, \\ 9.99 \times \texttt{weight} + 625 \times \texttt{height} - 4.92 \times \texttt{age\_yrs} - 161 & \text{if } \texttt{sex} = 2. \end{cases} \tag{13}$$

Enrgy intake change was obtained via the identity:

$$\Delta EI^{(i)} = Cinit + \Delta \text{cal}^{(i)} \tag{14}$$

where initial calories are estimated by:

$$Cinit = RMR^{(i)} \times \text{PAL} \tag{15}$$

Initial fat mass was obtained via the function:

$$F(0) = \begin{cases} (1/100) \times \texttt{weight} \times (0.14 \times \texttt{age\_yrs} + 37.31 \times \ln(\texttt{bmi}) - 103.94) & \text{if } \texttt{sex} = 1, \\ (1/100) \times \texttt{weight} \times (0.14 \times \texttt{age\_yrs} + 39.96 \times \ln(\texttt{bmi}) - 102.01) & \text{if } \texttt{sex} = 2. \end{cases} \tag{16}$$

which allowed us to estimate initial lean mass using the initial `weight` variable.

Additional information on the model can be found in [5, 6, 7]

1.4 Algorithm

Estimation of body weight as in (2) was programmed into an `R` function called `BodyWeightChange` which required as input the individual's age, weight, height, energy intake change, sodium change and the length of the approximation (for how many days should the model run). `BodyWeightChange` used the `deSolve` package [8] for approximating the system of differential equations in (6). In particular we chose the numeric solver `lsoda` as it automatically determines whether to use *stiff* or *non-stiff* methods for the approximation. Numerical results were consistent with the ones developed at `https://www.supertracker.usda.gov/bwp/index.html` which use Rungue-Kutta of 4th order.

The pseudocode in 1 explains the procedure followed.

Algorithm 1 Weight change estimation

```
1: for  i ∈ Individuals in ENSANUT do
2:     NewWeight^(i) ← BodyWeightChange(age_yrs^(i), weight^(i), height^(i), sex^(i), Δ cal^(i), Δ na^(i), 3650)
3: end for
```

After weight change was estimated for each individual, we used the `survey` package (as specified above) to create summary statistics. The complete dataset with weight change for each individual can be found at the Open Science Framework `https://osf.io/khkwp/#` as `Adults_Hall.rda`.

2 Diabetes model

We developed Multi-cohort Population Markov State-Transition Models [9] of diagnosed diabetes in Mexico similar to those developed by [10, 11, 12] for the US population. Separate models for male and females were constructed, breaking the population into 101 age-classes (single years from $0 - 100$) and two disease-states (non-diabetic and with diagnosed-diabetes) for each gender.

For each gender, the number of non-diabetics ($NDiab$), diabetics ($Diab$) and new deaths ($Deaths$) in Year $t+1$ ($t = 2010, \ldots, 2049$) and age $A+1$ ($A = 0, \ldots, 99$) is given by:

$$\begin{aligned} NDiab(A+1, t+1) &= (1 - I(A,t) - D(A,t)) * NDiab(A,t) \\ Diab(A+1, t+1) &= I(A,t) * NDiab(A,t) * -RR * D(A,t) * Diab(A,t) \\ Deaths(A+1, t+1) &= D(A,t) * (NDiab(A,t) + RR * Diab(A,t)), \end{aligned} \tag{17}$$

Where $I(A,t)$ denotes the annual incidence of diagnosed diabetes for individuals of age A in Year t; $D(A,t)$ denotes the mortality rates for non-diabetics at age A in Year t, and RR denotes the mortality relative risk of diabetics versus non-diabetics. We assume that all infants are born without diabetes, i.e., $Diab(0, \cdot) = 0$.

We used estimates of diabetes 2010 prevalence from [13] as initial condition and then evolved the models using estimates of age-specific incidence of diagnosed diabetes $I(A, \cdot)$ as described below, Mexico's 2010 age-specific mortality rates (keeping these constant into the future), and projections of the number of births per year during $2010 - 2050$ ($ND(0,t)$, $t = 2010, ..., 2050$).

We assumed three scenarios for the baseline age-specific incidence of diagnosed diabetes, $D(A,t)$. The optimistic scenario assumes that future incidence will be similar to that of 2000, the middle-scenario assumes that future incidence will be equal to that of 2005, and the pessimistic-scenario assumes the 2010 incidence. To account for the differential mortality between individuals with and without a diabetes diagnosis, we assumed a relative risk of mortality, RR, of 2.0 for people with a diabetes diagnosis, consistent with estimates in the US in the 1990s and early 2000s, and with earlier Markov models of diabetes in the US [10, 12]. Sensitivity analyses assumed mortality relative risks of 1.5 and 3.0.

Diabetes and SSB consumption were linked using the meta-analytical relative risk (RR) of 1.26 per SSB serving (RR 1.26, 95%CI 1.12, 1.41, per 355 ml/serving) estimated by [14]. This RR directly links SSB consumption to diabetes risk, including both direct and weight-mediated effects. Under the tax scenarios, the population age-specific incidence of diagnosed diabetes, $I(A,t)$, was multiplied by a reduction factor:

$$RedFactor = exp(-log(1.26) * redml/355), \tag{18}$$

Where $redml$ represents the average reduction in consumption in milliliters by age and gender. We assumed that the tax went into effect in 2014, and a two year lag between implementation and the effects on diabetes risk.

3 Weight change model sensitivity analysis

In addition to the weight change model we also conducted a sensitivity analysis to see how the results would vary had we considered changing weight linearly using Relative Risks (RR) from both observational and experimental studies that allow substitution. For that purpose we compared the reduction in BMI from our

model with the ones resulting from applying RRs from other studies (in particular from [15]) and found the corresponding BMI from our model closely matched other BMI for metanalytic results. That is: under a different modeling strategy which allows substitution, we would expect roughly the same BMI changes. See Appendix Figure S6.

References

[1] Instituto Nacional de Salud Pública. Encuesta nacional de salud y nutrición. `http://ensanut.insp.mx/`, 2012. Online; accessed 24 May 2016.

[2] Thomas Lumley et al. Analysis of complex survey samples. *Journal of Statistical Software*, 9(1):1–19, 2004.

[3] Carson C Chow and Kevin D Hall. The dynamics of human body weight change. *PLoS Comput Biol*, 4(3):e1000045, 2008.

[4] Mark D Mifflin, Sachiko T St Jeor, Lisa A Hill, Barbara J Scott, Sandra A Daugherty, and YO Koh. A new predictive equation for resting energy expenditure in healthy individuals. *The American journal of clinical nutrition*, 51(2):241–247, 1990.

[5] Kevin D Hall, Gary Sacks, Dhruva Chandramohan, Carson C Chow, Y Claire Wang, Steven L Gortmaker, and Boyd A Swinburn. Quantification of the effect of energy imbalance on bodyweight. *The Lancet*, 378(9793):826–837, 2011.

[6] Kevin D Hall. Predicting metabolic adaptation, body weight change, and energy intake in humans. *American Journal of Physiology-Endocrinology and Metabolism*, 298(3):E449–E466, 2010.

[7] Kevin D Hall and Peter N Jordan. Modeling weight-loss maintenance to help prevent body weight regain. *The American journal of clinical nutrition*, 88(6):1495–1503, 2008.

[8] Karline Soetaert, Thomas Petzoldt, R Woodrow Setzer, et al. Solving differential equations in r: package desolve. *Journal of Statistical Software*, 33(9):1–25, 2010.

[9] Uwe Siebert, Oguzhan Alagoz, Ahmed M Bayoumi, Beate Jahn, Douglas K Owens, David J Cohen, Karen M Kuntz, ISPOR-SMDM Modeling Good Research Practices Task Force, et al. State-transition modeling: a report of the ispor-smdm modeling good research practices task force-3. *Value in Health*, 15(6):812–820, 2012.

[10] KM Venkat Narayan, James P Boyle, Theodore J Thompson, Stephen W Sorensen, and David F Williamson. Lifetime risk for diabetes mellitus in the united states. *Jama*, 290(14):1884–1890, 2003.

[11] Amanda A Honeycutt, James P Boyle, Kristine R Broglio, Theodore J Thompson, Thomas J Hoerger, Linda S Geiss, and KM Venkat Narayan. A dynamic markov model for forecasting diabetes prevalence in the united states through 2050. *Health care management science*, 6(3):155–164, 2003.

[12] James P Boyle, Theodore J Thompson, Edward W Gregg, Lawrence E Barker, and David F Williamson. Projection of the year 2050 burden of diabetes in the us adult population: dynamic modeling of incidence, mortality, and prediabetes prevalence. *Population health metrics*, 8(1):1, 2010.

[13] Rafael Meza, Tonatiuh Barrientos-Gutierrez, Rosalba Rojas-Martinez, Nancy Reynoso-Noverón, Lina Sofia Palacio-Mejia, Eduardo Lazcano-Ponce, and Mauricio Hernández-Ávila. Burden of type 2 diabetes in mexico: past, current and future prevalence and incidence rates. *Preventive medicine*, 81:445–450, 2015.

[14] Vasanti S Malik, Matthias B Schulze, and Frank B Hu. Intake of sugar-sweetened beverages and weight gain: a systematic review. *The American journal of clinical nutrition*, 84(2):274–288, 2006.

[15] Vasanti S Malik, An Pan, Walter C Willett, and Frank B Hu. Sugar-sweetened beverages and weight gain in children and adults: a systematic review and meta-analysis. *The American journal of clinical nutrition*, 98(4):1084–1102, 2013.
